# Effects of environmental factors and appendage injury on the wing variation in the cricket *Velarifictorus ornatus*

**DOI:** 10.1093/jis/14.1.117

**Published:** 2014-09-01

**Authors:** Lü-Quan Zhao, Dao-Hong Zhu

**Affiliations:** 1 Laboratory of Insect Behavior & Evolutionary Ecology, Central South University of Forestry and Technology, Changsha, Hunan 410004, China; 2 Laboratory of Zoology, Hunan First Normal University, Changsha, Hunan 410205, China

**Keywords:** wing dimorphism, Gryllidae, photoperiod, density, injury

## Abstract

The effects of environmental factors and appendage injury on the wing variation in *Velarifictorus ornatus* (Shiraki) (Orthoptera: Gryllidae) were investigated. The percentage of micropters was more than 95% when the nymphs were reared at constant photoperiods, and changing photoperiod did not affect wing variation in *V. ornatus* at 25 or 30°C. In the crowding experiment, the percentage of macropters was only 11.2% when the nymphs were reared separately at 25°C. In contrast, the percentage of macropters was significantly higher when the rearing density was increased to two nymphs per container and lower when the rearing density was increased to five or 10 nymphs per container. These results indicate that low and high rearing densities induce micropters, but intermediate rearing density stimulates the formation of macropters. Meanwhile, severance of appendages, such as antennae, femora, and tibiae, in the nymph stage exerted a micropterizing effect. The period sensitive to such stresses ranged from 35 to 60 days of nymph development.

## Introduction

Wing dimorphism, occurring in Orthoptera, Psocoptera, Thysanoptera, Homoptera, Heteroptera, Coleoptera, and Hymenoptera, is a widespread phenomenon in insects ( Zera and Denno 1997 ). In general, two morphologically distinct morphs exist: long-winged and short-winged. The long-winged morph, also called the macropterous morph, is normally able to fly, whereas the short-winged morph, often referred to as the micropterous or brachypterous morph, cannot fly. In some species (such as aphids), the flightless morph lacks wings and is called the apterous morph ( Tanaka et al. 2001 ).

Environmental factors, such as crowding, photoperiod, temperature, and food, influence theproportion of the two wing forms ( Zera and Denno 1997 ). Macropters are primarily responsible for escaping deteriorating habitats and colonizing new ones ( Roff 1986 , Roff and Fairbairn 1991 ). Thus, high crowding, food shortage, and poor food quality induce the macropterous morph that can disperse to new habitats ( Dai et al. 1997 ). For instance, the incidence of macroptery in *Allonemobius so-cius* (Scudder) and *Velarifictorus asperses* (Walker) (Orthoptera: Gryllidae) increased with increasing density ( Olvido et al. 2003 , Zeng et al. 2010 ). The same was shown for *Dimorphopterus japonicus* Hidaka (Heteroptera: Lygaeidae) and *Eobiana engelhardti* Uvarov (Orthoptera: Tettigoniidae) ( Sasaki et al. 2002 , Nakao 1999). In *Gryllus rubens* Scudder (Orthoptera: Gryllidae), when the nymphs were individually reared, the incidence of micropters was only 13%, but it increased to 43% when the nymphs were group reared ( Zera and Tiebel 1988 ). Furthermore, long-winged forms could be redirected to short-winged forms by transferring nymphs from individual to group rearing as late as the last nymphal stadium. That means high density induced more brachypterous morphs than low density in *G. rubens* ( Zera and Tiebel 1988 ). The increased production of the short-winged phenotype when insects were reared in groups is unusual. But, there are no reports that offer explanations for a brachypterizing effect of group rearing in wing-dimorphic insects

In addition to environmental factors, stresses also affect wing variation in the wing dimorphism of insects. For example, removal of a hind wing pad from late-instar nymphs of *Allonemobius fasciatus* (De Geer) (Orthoptera: Gryllidae) inhibited the formation of long-winged adults ( Tanaka 1985 ). Shimizu and Masaki (1993) reported that severance of appendages or exposure to heat or cold in the nymph stage of *Dianemobius fascipes* (Walker,) (Orthoptera: Gryllidae) exerted a micropterizing effect.

**Figure 1.**
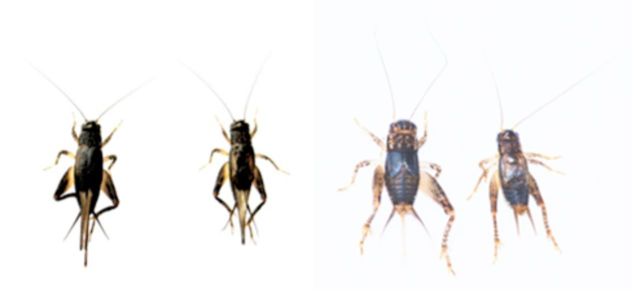
Adults of *V. ornatus.* From the left, macropterous female, macropterous male, brachypterous female, and brachypterous male. High quality figures are available online

The cricket *Velarifictorus ornatus* (Shiraki) (Orthoptera: Gryllidae) is widespread in China ( Yin and Liu 1995 ). Adults have either fully developed wings (macropterous) or reduced wings (brachypterous) ( Fig. 1 ). Our preliminary experiments suggested that constant and changing photoperiods do not affect wing variation. Low and high rearing density induced micropters, but intermediate rearing density stimulated the production of macropters. The purpose of this study was to clarify relationships between each of these environmental factors and the incidence of macroptery by climate-controlled experiments and to determine the effects of various injuries on wing variation in *V. ornatus* by means of severance of appendages in the nymph stage.

## Materials and Methods

### Insects

The insects were derived from a laboratory colony originally collected in Zhuzhou (27° 48' N, 113° 12' E), China, in May 2005. Nymphs and adults were reared according to a method described by Zhao et al. (2008 , 2010).

### Effect of photoperiod on wing variation in *V. ornatus*

To investigate the effect of different constant photoperiods on wing variation in *V. ornatus,* newly hatched individuals were randomized and reared at 25°C and a photoperiod of 16:8 (L:D), 14:10 (L:D), or 12:12 (L:D) and at 30°C and a photoperiod of 16:8 (L:D) or 12:12 (L:D). Wing form, according to the length of hind wings, was checked and recorded every day after adults began to emerge.

The duration of nymph development of *V. ornatus* was very long (> 200 days) under constant photoperiods at 25°C. As shown previously, it was prolonged further when nymphs were transferred from long days to short days, but was shortened significantly in the reverse transfer (i.e., from short days to long days) ( Zhao et al. 2008 ). Hence, we designed the following experiments to investigate the influence of a changing photoperiod on wing variation in *V. ornatus.* Newly hatched nymphs were assigned randomly to four different types of treatments that varied in the day length and the timing of the shift. The short-day photoperiod was 12:12 (L:D), the intermediate-day photoperiod was 14:10 (L:D), and the long-day photoperiod was 16:8 (L:D). In treatment 1, nymphs were maintained at 25°C and subjected to a shift in photoperiod from relatively shorter to longer days. The transfer from the short- to long-day photoperiod was performed after 10, 30, 60, and 90 days; that from the short- to intermediate-day photoperiod after 60 days; and that from the intermediate- to long-day photoperiod after 30 and 60 days. In treatment 2, nymphs were maintained at 25°C and subjected to a shift in photoperiod from relatively longer to shorter days. The transfer from the long- to short-day photoperiod was performed after 30, 60, and 90 days; that from the long-to intermediate-day photoperiod after 60 days; and that from the intermediate- to short-day photoperiod after 30 and 60 days. In treatment 3, nymphs were maintained at 30°C and subjected to a shift in photoperiod from short to long days after 10, 20, 50, and 80 days. In treatment 4, nymphs were maintained at 30°C and subjected to a shift in photoperiod from long to short days after 10, 30, and 50 days. All experimental groups were checked daily for adult emergence, and the short- and long-winged individuals were recorded.

### Effect of rearing density on wing variation in *V. ornatus*

The results showed that the incidence of macropters was very low at 25°C ( Table 1 ). Thus, the long-winged males were paired with long-winged females to raise the incidence of macropters in the offspring. The nymphs (F1 generation) from these adults were used to elucidate the effect of rearing density on wing variation in *V. ornatus* . Newly emerged nymphs (F _1_ generation) were reared at densities of one, two, five, and 10 individuals per container (13.0 × 13.0 × 8.5 cm). The number of replicates for each density was 40 (one individual), 20 (two individuals), 10 (five individuals), and seven (10 individuals). To shorten the period of nymph development ( Zhao et al. 2008 ), nymphs were kept first at 25°C and a photoperiod of 12:12 (L:D) for 30 days and then transferred to 25°C and a photoperiod of 16:8 (L:D).

**Table 1.**
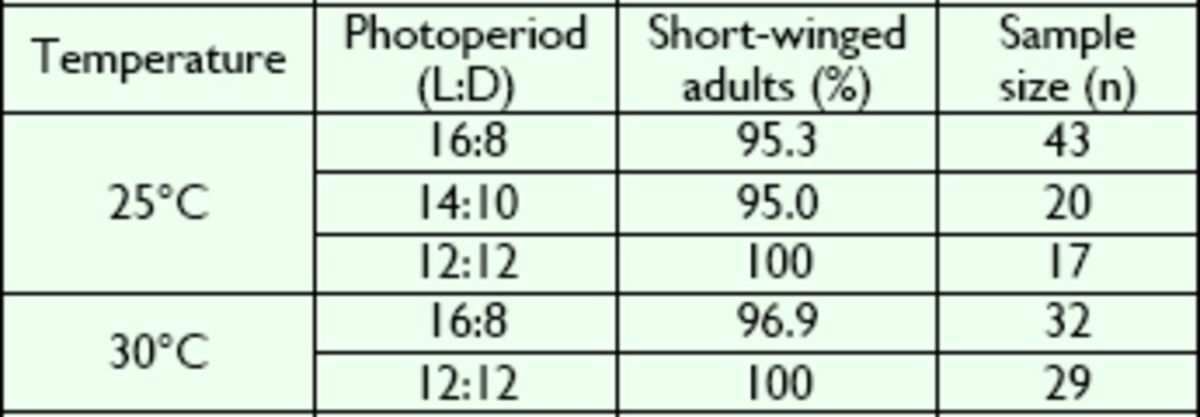
Effect of constant photoperiod on the wing variation in *V. ornatus* at 25°C and 30°C.

No significant difference was found between the percentages of short-winged adults at 25°C ( *P* > 0.05; Kruskal-Wallis test) and between those at 30°C ( *P* > 0.05; Wilcoxon test).

### Effect of appendage injury on wing variation in *V. ornatus*

*Velarifictorus* crickets often fight, causing the loss of appendages. To investigate the relationship between appendage injury and wing variation in *V. ornatus* , newly emerged individuals were reared singly. After the development of nymphs to penultimate instars, one antenna or two antennae were cut using a fine forceps. In other individuals, the meso-femora, meso-tibiae, or entire middle legs (below the coxa) were amputated by using dissecting scissors. The nymphs were anesthetized on ice to facilitate the operation and decrease the inflicted sensation. The control nymphs were only anesthetized without undergoing any operation. After the operation, the nymphs were reared singly. They were reared first at 25°C and a photoperiod of 12:12 (L:D) h for 30 days and then transferred to 25°C and a photoperiod of 16: 8 (L:D) to shorten the period of nymph development ( Zhao et al. 2008 ). The short- and long-winged individuals were recorded after adult emergence. The nymphs used in this experiment were the F _3_ generation reared from long-winged adults.

### Identification of the sensitive phase during which appendage injury causes wing variation in *V. ornatus*

Based on the results obtained in the experiments described before, rearing conditions in this experiments were 25°C at a photoperiod of 12:12 (L:D) for 30 days, after which nymphs were transferred to 25°C at a photoperiod of 16:8 (L:D). At this regime, nymphs developed to the penultimate instars within 45 days and to the last instars within 60 days. To identify the sensitive phase during which appendage injury would cause wing variation, the middle legs of nymphs were removed during cold-anesthesia at 25, 35, 45, and 60 days. Control nymphs were cold-anesthetized but not injured. The short- and long-winged individuals were recorded after adult emergence. The nymphs used in this experiment were the F _4_ generation reared from long-winged adults.

### Statistical analysis

Data between more than two groups that failed the equal variance and normality tests were subjected to the Kruskal-Wallis test. The Wilcoxon test was used to compare the data between two groups. All data were statistically analyzed using SPSS 13.0 (IBM, www.ibm.com ). In all cases, the significance level α was 0.05.

## Results

### Effect of photoperiod on wing variation in *V. ornatus*

All the adults were short-winged individuals at 25°C and a photoperiod of 12:12 (L:D) ( Table 1 ). At the same temperature, the percentage of microptery was 95.0% and 95.3% at a photoperiod of 14:10 (L:D) and 16:8 (L:D), respectively ( Table 1 ). However, these differences were not statistically significant ( *P* > 0.05; Kruskal-Wallis test; Table 1 ). At 30°C, no obvious differences in the incidence of microptery were observed between photoperiods of 12:12 (L:D) and 16:8 (L:D) ( *P* > 0.05; Wilcoxon test; Table 1 ). These results indicate that different constant photoper-photoperiods did not affect the wing variation in *V. ornatus* .

The photoperiod naturally changes with seasonal change; thus, the effect of shifts in photoperiod on wing variation was investigated. At 25°C, the newly emerged nymphs were first reared at a photoperiod of 12:12 (L:D) for 10, 30, 60, and 90 days, before transfer to 16:8 (L:D). The percentage of macroptery in these treated groups was higher than that in the groups held at constant photoperiods. Furthermore, the percentage of macroptery tended to be related to the timing of the change; the incidence of macroptery increased when the photoperiod was changed at an early stage. However, no statistical significance was found ( *P* > 0.05; Kruskal-Wallis test; Table 2 ). No mactropterous adults emerged when nymphs were transferred from a photoperiod of 12:12 (L:D) to a photoperiod of 16:8 (L:D) after 90 days ( Table 2 ). All other shifts from relatively shorter to longer photoperiods (within 30 or 60 days) yielded a slightly higher percentage of macropterous adults than the maintenance of nymphs at constant photoperiods ( Tables 1 and 2 ). Reverse transfer (from long to short days) yielded 100% short-winged adults regardless of the timing and amplitude of the shift in photoperiod ( Table 3 ). At 30°C, similar results were obtained when the photoperiod was changed from short to long days or vice versa; the percentage of short-winged adults ranged from 82.4 to 100% with no statistically significant differences ( *P* > 0.05; Kruskal-Wallis test; Table 4 ). These results showed that a shift in photoperiod did not affect wing variation in *V. ornatus* at 25 or 30°C.

**Table 2.**
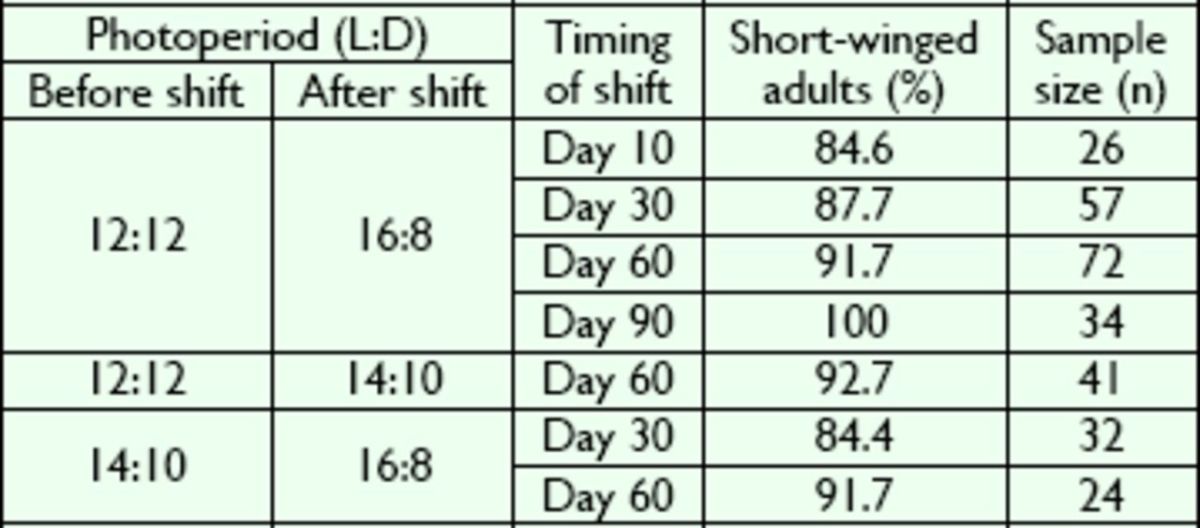
Effect of photoperiodic shift from short to long days on the wing variation in *V. ornatus* at 25°C.

No significant difference was found between the percentages of short-winged adults ( *P* > 0.05; Kruskal-Wallis test).

**Table 3.**
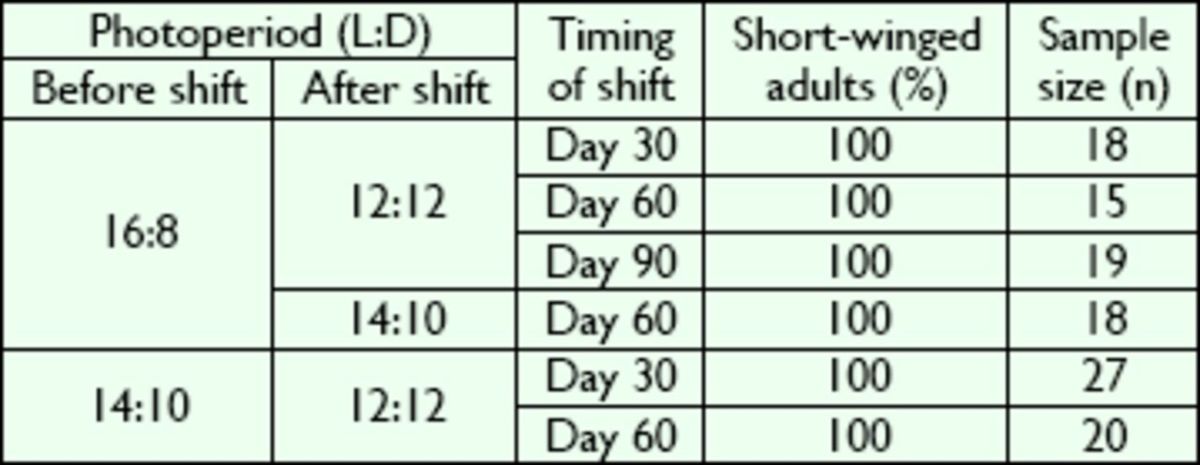
Effect of photoperiodic shift from long to short days on the wing variation in *V. ornatus* at 25°C.

No significant difference was found between the percentages of short-winged adults ( *P* > 0.05; Kruskal-Wallis test).

**Table 4.**
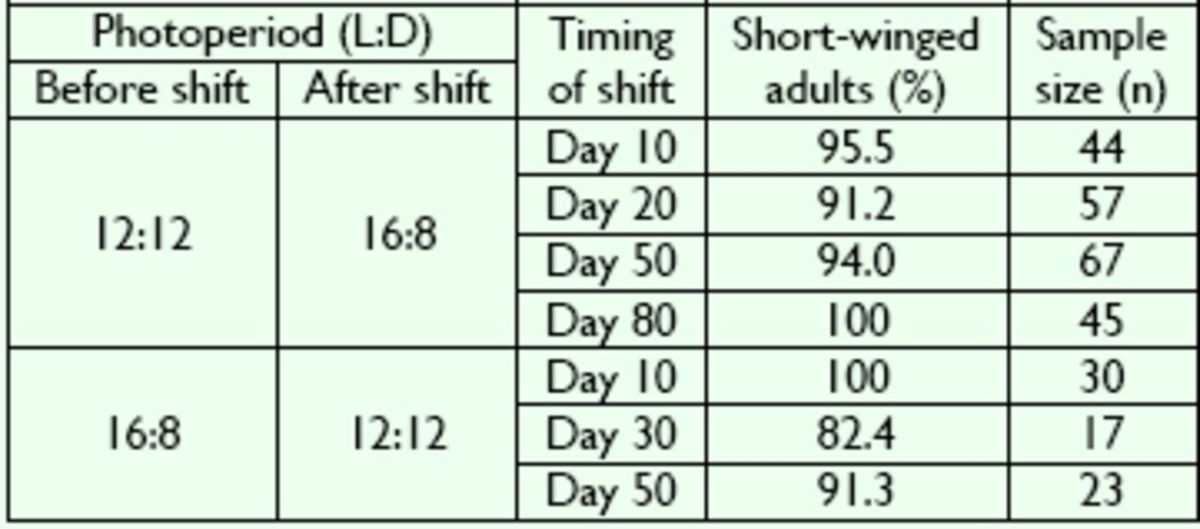
Effect of photoperiodic shift on the wing variation in *V. ornatus* at 30°C.

No significant difference was found between the percentages of short-winged adults ( *P* > 0.05; Kruskal-Wallis test).

### Effect of rearing density on wing variation in *V. ornatus*

**Figure 2.**
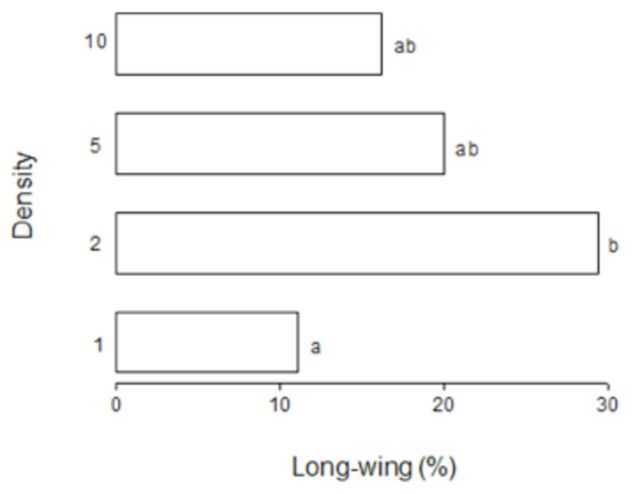
Effect of crowding on wing morphs of the cricket *V. ornatus* (density = 1, n = 27; density = 2, n = 36; density = 5, n = 45; density = 10, n = 64). Bars labeled with the same letters are not significantly different ( *P* > 0.05), whereas bars labeled with different letters are significantly different ( *P* < 0.05; Kruskal-Wallis test). High quality figures are available online.

Figure 2 shows the incidence of macroptery at various rearing densities. This incidence was 11.2% when the nymphs were reared singly. The percentage of macroptery increased significantly to 29.4% a t a density of two nymphs per container ( *P* < 0.05; Wilcoxon test; Fig. 2 ). However, the percentage of macropterous adults decreased to 20.4% and 16.1% when density was increased to five and 10 nymphs per container, respectively. The percentages of macropters at these densities did not differ significantly from those observed when nymphs were held singly or at two individuals per container ( *P* > 0.05; Krus-kal-Wallis test; Fig. 2 ). These results showed that, compared with single maintenance, a low density of two individuals per container induced the formation of macropterous adults, whereas a high density of five or 10 individuals per container did not.

### Effect of appendage injury on wing variation in *V. ornatus*

**Figure 3.**
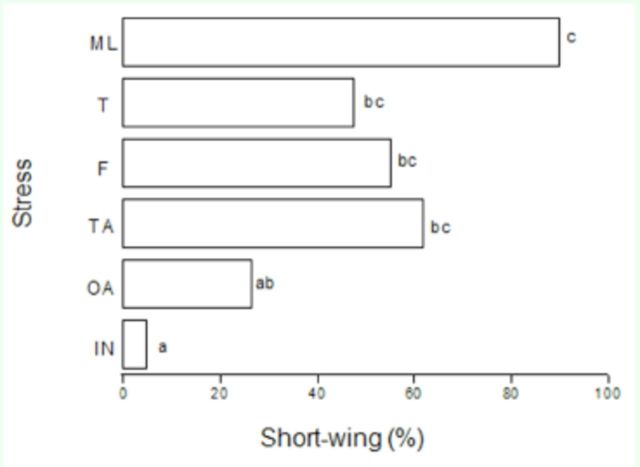
Effect of stress inflicted by surgical removal of appendages on the wing morph of the cricket *V. ornatus* (ML, middle legs; T, tibiae; F, femora; TA, two antennae; OA, one antenna; IN, intact). Bars labeled with the same letters are not significantly different ( *P* > 0.05), whereas bars labeled with different letters are significantly different ( *P* < 0.05; Kruskal-Wallis test; n = 20-30). High quality figures are available online.

Figure 3 shows the micropterizing effects of severed antennae, meso-femora, meso-tibiae, and entire middle legs inflicted on the penultimate nymphal instar. All treatments, except for the removal of one antenna, suppressed the development of long wings significantly ( *P* < 0.05; Kruskal-Wallis test, Fig. 2 ).

### Sensitive phase during which appendage injury causes wing variation in *V. ornatus*

**Figure 4.**
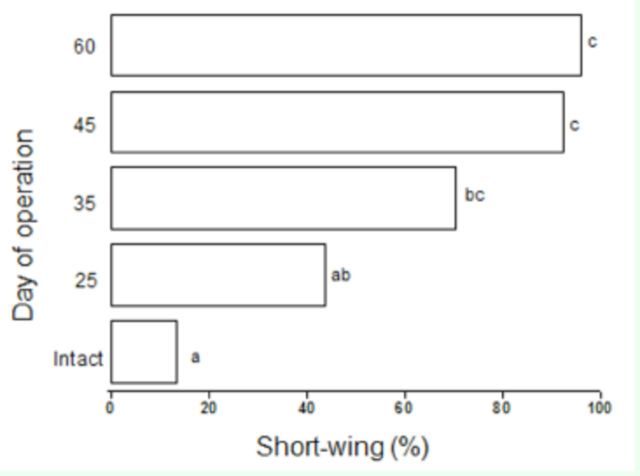
Effect of middle leg loss on the wing morph of the cricket *V. ornatus.* Bars labeled with the same letters are not significantly different ( *P* > 0.05), whereas bars labeled with different letters are significantly different ( *P* < 0.05; Kruskal-Wallis test; n = 20-30). High quality figures are available online.

The middle legs of nymphs were removed at 25, 35, 45, and 60 days after hatching. When the operation was performed at 25 days, the incidence of micropterous adults was similar to that in non-injured individuals ( Fig. 4 ). However, when the operations were performed at 35, 45 or 60 days, the incidence of micropters was significantly higher than that in non-injured individuals; furthermore, the incidence of micropters was significantly higher when the middle legs were removed at 45 and 60 days than when they were removed at 25 days *(P* < 0.05; Kruskal-Wallis test; Fig. 4 ). These results indicate that the penultimate and last nymphal instars were most sensitive to injury as indicated by the formation of short-winged individuals.

## Discussion

Various environmental cues, such as crowding, host plant condition, temperature, and photoperiod, affect insect wing form ( Zera and Denno 1997 ). For example, in *Cavelerius saccharivorus* Okajima (Heteroptera: Lygaeidae), more macropters were produced under long photoperiods, high temperatures, and high densities than under the opposite conditions ( Murai 1975 ; Fujisaki 1986 , 1989). In *Pyrrhocoris sibiricus* Kuschakevich (Heteroptera: Pyrrhocoridae), high temperature, short daylength, and moderate crowding were favorable conditions for macropter production ( Sakashita et al. 1995 ). In contrast, the wing form in *Pteronemobius taprobansis* Walker (Ortheoptera: Gryllidae) was mainly influenced by photoperiodic change; macropterous adults were few under constant long-day and short-day photoperiods ( Tanaka et al. 1976 ). Thus, the environmental factors responsible for the production of macropters vary between species.

The present results indicate that photoperiod does not influence the wing form in *V. ornatus.* Mostly micropters were produced under long, intermediate, short, or changing photoperiods. However, results from studies on *Oxya yezoensis* Shiraki (Orthoptera: Acrididae) ( Zhu 2001 ) and *D. japonicus* ( Sasaki et al. 2002 ) were different, showing that long photoperiod induced development of macropters in both species. Wing variation in the cricket *P. taprobanensis* was not affected by constant long- or short-day photoperiods, but shifts in photoperiod could induce macropterous forms ( Tanaka et al. 1976 ). Alexander (1961 , 1968) suggested that environmental factors, such as high temperature, high density, and eliminated diapause, shortened the nymphal stadium and induced the production of macropters. *Velarifictorus ornatus* overwinter as nymphs, and our results showed that changing photoperiods accelerated nymph development but did not promote the formation of long wings.

The ability to disperse by flight is an important feature of insects that has played a key role in their evolutionary success ( Roff and Fairbairn 1991 , Wagner and Liebherr 1992 ). Thus, wing dimorphic adults often become long-winged morphs when the nymphs are reared at high density or on nutritionally inadequate food. The present results showed that the incidence of macropters in *V. ornatus* increased with increasing density (from one to two individuals per container). However, when the density was increased to five or 10 individuals, the incidence of macropters was similar to that observed with one nymph per container. The increase in density from one to two individuals made body contact possible. When the density increased to five or 10 individuals, body contact likely became more frequent, and competition between individuals likely increased. We therefore hypothesized that this situation would favor the production of macropters to escape an unfavorable habitat. However, when the density became very high, fighting between individuals may have caused shedding of appendages and promoted the formation of short-winged individuals. For example, when appendages (hind femora, hind tibiae, etc.) in the cricket *D. fascipes* were cut off during the nymph stage, this injury directed the development from long to short wings in the presumptive macropters ( Shimizu and Masaki 1993 ). Our results showed that cutting the appendages of nymphs of *V. ornatus* also had a micropterizing effect and support the notion that the low incidence of macropters at densities of five and 10 individuals per container could be a result of appendage loss from fighting. The period of nymphs’ sensitivity to appendage loss, as indicated by the observed micropterizing effect, ranged from 35 to 60 days after hatch and was most significant between 45 and 65 d when nymphs had reached the penultimate and last instar, respectively. These results are in accordance with those reported for the cricket *D. fascipes* ( Shimizu and Masaki 1993 ).
